# Development of MVA-d34 Tetravalent Dengue Vaccine: Design and Immunogenicity

**DOI:** 10.3390/vaccines11040831

**Published:** 2023-04-12

**Authors:** Ramil R. Mintaev, Dina V. Glazkova, Olga V. Orlova, Georgiy M. Ignatyev, Alexey S. Oksanich, German A. Shipulin, Elena V. Bogoslovskaya

**Affiliations:** 1 Federal State Budgetary Institution “Centre for Strategic Planning and Management of Biomedical Health Risks” of the Federal Medical Biological Agency, 119833 Moscow, Russia; 2 I. Mechnikov Research Institute of Vaccines and Sera, 105064 Moscow, Russia

**Keywords:** dengue, vaccine, MVA

## Abstract

Dengue fever, an infectious disease that affects more than 100 million people every year, is a global health problem. Vaccination may be the most effective prevention strategy for the disease. However, the development of vaccines against dengue fever is complicated by the high risk of developing an antibody-dependent increase in infection. This article describes the development of an MVA-d34 vaccine against the dengue virus based on a safe and effective MVA viral vector. The DIII domains of the envelope protein (E) of the dengue virus are used as vaccine antigens, as antibodies against these domains do not cause an enhancement of infection. The use of the DIII domains of each of the four dengue virus serotypes made it possible to generate a humoral response against all four dengue virus serotypes in immunized mice. We also showed that the sera of vaccinated mice present virus-neutralizing activity against dengue serotype 2. Thus, the developed MVA-d34 vaccine is a promising candidate vaccine against dengue fever.

## 1. Introduction

Dengue fever is a viral disease that occurs in tropical and subtropical regions of the world. Approximately 390 million cases of the disease are recorded annually, with 3 billion people remaining at risk of infection. The cause of the disease is a virus belonging to the Flaviviridae family, which is transmitted by mosquitoes of the genus *Aedes* [1]. There are four known serotypes of the dengue virus (DENV). The immune response against one serotype shows a cross-reactive effect against other serotypes, but with incomplete neutralization of the virus [2]. Moreover, upon infection with a heterologous serotype, previously acquired immunity has the effect of enhancing the infection, with possibly fatal consequences. These antigenic features of dengue viruses complicate the development of an effective vaccine.

Currently, one licensed tetravalent Dengvaxia vaccine based on a yellow fever virus vaccine variant has been reported. In clinical studies, the protective efficacy of this vaccine has been shown to differ for different dengue virus serotypes, with rates of 61.2%, 81.9%, and 90% against serotypes 1, 3, and 4, respectively [3]. The vaccine does not protect against dengue virus serotype 2. The main disadvantage of this vaccine is the danger of vaccination for people who have not previously had dengue fever. It has been demonstrated that in this case, the postvaccination infection of a person with dengue fever virus can develop an antibody-dependent enhancement of infection (ADE), which leads to a more severe course of the disease, sometimes with a fatal outcome [4]. Children are most affected by this phenomenon. Thus, an 8-fold increase in cases of hospitalization among patients with severe dengue fever was observed in children 2–5 years of age within 3 years after immunization [3,4,5]. Therefore, this vaccine is currently recommended only for people who have previously had dengue fever (seropositive individuals), because only in this case is the vaccine considered safe.

In this context, the search for new approaches for the development of a safe and effective vaccine against all serotypes of the dengue virus is still important. The E surface protein of the dengue virus is one of the main targets of the humoral response; however, only antibodies against the DIII domain of the E protein provide high neutralizing activity without the effect of increasing infection [6,7]. Previously, several variants of vaccines based on the DIII domain have been proposed. DNA vaccines [8,9], recombinant proteins [10], virus-like particles [11], and a vector vaccine based on the measles virus [12] were used as platforms in such studies. However, none of these candidate vaccines has been approved for use to date.

The modified vaccinia virus Ankara (MVA) is an attractive vaccine vector due to its high safety profile demonstrated in a large human population and its ability to stimulate an immune response even in immunocompromised people [13]. The aim of this work was to develop a vaccine against all four serotypes of the dengue virus based on the MVA viral vector.

## 2. Materials and Methods

### 2.1. Cell Lines and Viruses

A modified vaccinia virus, Ankara (MVA), was obtained from the American Type Culture Collection (ATCC^®^ VR-1566). Later in the article, the variant will be referred to as the MVA or wt MVA (indicating that it is not a recombinant). The MVA virus was replicated using the Syrian hamster fibroblast cell line BHK-21, which was cultivated in DMEM containing 10% fetal bovine serum (FBS), 4 mM L-glutamine (Gibco, Big Cabin, OK, USA), 1 mM sodium pyruvate (Gibco, USA), and 10 mM HEPES (Gibco, USA) at 37 °C and 5% CO_2_. Amplification and the calculation of virus titer (TCID50) was performed according to the methods described previously [14,15]. HEK293FT cells (ATCC human embryonic kidney cells, CRL-11268) were cultured in DMEM containing 10% FBS, 4 mM L-glutamine (Gibco, United States), and 1 mM sodium pyruvate (Gibco, United States) at 37 °C in a 5% CO_2_ atmosphere.

The *Aedes albopictus* C6/36 (ATCC CRL-1660) mosquito cell line was cultured on Leibovitz L-15 medium supplemented with 10% fetal serum (Gibco) at 32 °C with 5% CO_2_.

The dengue viruses of different serotypes used as antigens (type 1—Hawaii strain, type 2—New Guinea C (NGC), type 3—H-87, type 4—H241) were obtained from the State Collection of Viruses of II-IV Pathogenicity Groups N.F. Gamaleya of the Federal State Budgetary Institution N. F. Gamaleya National Research Center of the Ministry of Health of Russia. Serotype 2 dengue virus (DENV2) was obtained from *Aedes aegypti* mosquitoes in the population of Nicaragua, and the isolation and cultivation of DENV2 have been described previously [16].

### 2.2. Construction of Plasmids

**Figure A1:**
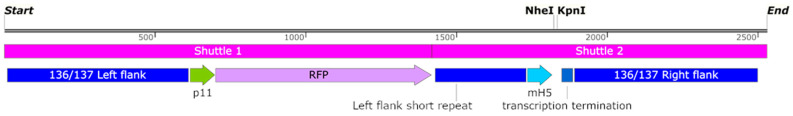
Map of the functional part of the vector pShuttle-136/137. Blocks labeled Shuttle 1 and Shuttle 2 show the division of the design into fragments, necessary for the synthesis of the sequence. p11 and mH5 promoters from the vaccinia virus drive the expression of RFP and GOI, respectively. The vaccinia virus termination signal (labeled as Term on the scheme) was used to stop the transcription of RFP gene and GOI. The DNA sequence of GOI can be inserted using restriction sites NheI/KpnI.

The dengue antigen d34 consisted of four DIII domains of the E protein, one for each of the four dengue virus serotypes, linked by a flexible linker, 2x(GGGGS) (Figure A1). The dengue virus genome records of serotypes 1 (record ID NP_05943), 2 (record ID NP_056776), 3 (record ID YP_001621843), and 4 (record ID NP_073286) were used to compile the sequence encoding the antigen. At the 5′-end of the open reading frame of the d34 gene, a sequence encoding the mouse Igk leader for directing the protein along the secretory pathway [17] was inserted, and the sequence encoding the 6xHis tag was located at the 3′-end.

The synthesis of the nucleotide sequences of the functional part of a shuttle vector and a sequence encoding the antigen was carried out by TOP Gene Technologies, Inc. (Quebec, QC, Canada), from which the following plasmids were obtained: pShuttle1, pShuttle2, and pUC16-d34.

The sequences from pShuttle1 and pShuttle2 were cloned into the pGEM-Easy plasmid (Promega, Madison, WI, USA) for plasmid shuttle vector generation (pShuttle-136/137). The plasmid shuttle vector contained the red fluorescent marker gene under the p11 MVA promoter, homology arms to the 136/137 MVA locus, the mH5 MVA promoter, vaccinia transcription termination signals, and NheI and KpnI restriction sites for the gene of interest cloning (Figure A1). The homology arms were identical to the nucleotide sequences 129,325–129,925 (left arm) and 129,926–130,532 (right arm) of the MVA genome (record GeneBank ID U944848). The cassette for RFP expression was additionally flanked with a short-left arm (MVA genome region: 129,626–129,925), which provided the marker gene removal by means of homologous recombination at the second stage of rMVA generation [14].

Additionally, to obtain the pShuttle-136/137-d34 plasmid, the d34 sequence was inserted into pShuttle-136/137 using NheI and KpnI restriction sites. Plasmid DNA was produced in E. coli cells (the DH5a strain) and isolated using the Plasmid Mini Kit (QIAGEN, Hilden, Germany) or Plasmid Maxi Kit (QIAGEN) under sterile conditions. The correctness of the construct was confirmed by Sanger sequencing.

### 2.3. Generation of Recombinant MVA

The recombinant MVA virus was obtained, purified, and produced as described previously [18]. During virus production, BHK21 cells were grown in DMEM containing 2% FBS, 4 mM L-glutamine, 1 mM sodium pyruvate, and 10 mM HEPES. Briefly, the cells were infected with wt MVA virus, and after 90 min, the growth medium was replaced with fresh medium, and transfection was performed with the pShuttle-136/137-d34 shuttle vector described in Section 2.2. After 48 h, the recombinant virus mixture was collected and plated to obtain individual plaques. Plaques containing the fluorescent marker were collected and reused for plaque preparation. The procedure was repeated until a virus free of wild virus contaminants was obtained. The absence of wild-type MVA in the recombinant virus was verified by PCR analysis with electrophoretic detection. A schematic representation of this process is shown in Figure S1. The average viral titer of wt MVA or rMVA-d34 stocks was 5 × 10^8^ pfu/mL.

### 2.4. Sera from Patients Immunocompetent for DENV, TBEV, and YFV

For ELISA and Western blotting, sera from dengue convalescent individuals (without specifying the serotype) or individuals vaccinated with the vaccine against tick-borne encephalitis (TBEV), produced by the Chumakov Federal Scientific Center for Research and Development of Immune-and-Biological Products of the Russian Academy of Sciences, or vaccinated with the yellow fever virus (YFV) vaccine, also produced by the Chumakov Federal Scientific Center for Research and Development of Immune-and-Biological Products of Russian Academy of Sciences, were used. Convalescent sera from DENV-infected individuals were obtained as part of a study of the prevalence of flavivirus-related fevers in Nicaragua [19]. The sera of vaccinated individuals were obtained by assessing the immune status of FMBA employees vaccinated with tick-borne encephalitis and yellow fever vaccines. Preliminary data on the presence/absence of IgG specific to DENV, TBEV, and YFV were confirmed by ELISA using the following kits: DENV—“Anti-Dengue Virus ELISA (IgG)” (EI 226b-9601G, Euroimmun AG, Germany); TBEV—“VektoVKE-IgG” (JSC “Vector-Best,” Russia); YFV—“Qualitative Human Yellow Fever Virus Antibody IgG (YFV-IgG) ELISA Kit” (MyBioSource Inc., San Diego, CA, USA).

### 2.5. Western Blotting

BHK-21 or HEK293FT cells were transduced with MVA virus and recombinant MVA-d34 virus at an MOI = 2 for 90 min. Cells without added viruses were used as a negative control. Then, the growth medium was replaced with serum-free OptiMEM. After 24 h, the cells were harvested, washed three times with phosphate-buffered saline (PBS), resuspended in RIPA lysis buffer containing 1% NP-40, and sonicated. Cellular debris was removed by centrifugation (15,000× *g*, 15 min, 4 °C). The culture supernatant was collected separately and concentrated using an Amicon Ultra0.5 centrifuge concentrator with a pore diameter of 10 kDa. The protein marker ECL Rainbow (Cytiva, Marlborough, MA, USA) or PageRuler Plus (Thermo Scientific, Waltham, MA, USA), and the samples were subjected to 12% SDS-PAGE. After electrophoresis under reducing conditions, the proteins were transferred to an Immun-Blot LF PVDF membrane (Bio-Rad, Hercules, CA, USA). The membranes were blocked in PBS containing 5% skimmed milk powder, ECL Prime Blocking Agent (PRN418, Amersham), and 0.1% Tween 20 and then incubated overnight at 4 °C with anti-6x-His Tag antibodies (HIS. H8- HRP, Invitrogen, USA) at a dilution of 1:20,000, along with human sera collected from dengue fever convalescent patients (without specifying the serotype) (serum 1, 1:1000) or people vaccinated with the TBEV vaccine (serum 2, 1:1000), TBEV and YFV vaccine (serum 3, 1:1000), or YFV vaccine (serum 4, 1:1000). Membranes hybridized with human sera were additionally incubated with goat anti-human IgG (H + L) HRP secondary antibodies at 1:30,000 (31410, Invitrogen, Waltham, MA, USA) for 1 h at room temperature. Then, the membranes were washed three times in PBS with 0.1% Tween 20. Protein complexes were determined using Clarity Western ECL Substrate (Bio-Rad, Hercules, CA, USA) according to the manufacturer’s recommendations and visualized using X-ray film (Fuji Film, Tokyo, Japan).

### 2.6. Experimental Animals

In order to study the antigenic properties of the experimental vaccine, 6- to 8-week-old mature female mice, hybrids of the first generation of the C57, and CBA lines, weighing 18–20 g, were used. The animals were obtained from the nursery of the Scientific Center for Biomedical Technologies, Russian Academy of Sciences, Russia.

### 2.7. Mice Immunization and Experimental Design

Five mice in the experimental vaccine group were immunized intramuscularly with 0.2 mL of the experimental vaccine (5 × 10^7^ MVA-d34 infectious particles), and five mice in the mock group were immunized intramuscularly with 0.2 mL wt MVA (5 × 10^7^ infectious particles).

Animals were immunized twice, with the second vaccine administered on day 21 after the first vaccination. On day 21 after the second immunization, serum samples were taken from mice in each group to determine antibody titers.

### 2.8. Preparation of the d34 Antigen for ELISA

HEK293FT cells were transduced with recombinant MVA-d34 virus at an MOI = 4 for 90 min. Then, the medium with the virus was replaced with serum-free OptiMEM. After 48 h, the culture supernatant was collected and clarified, and the protease inhibitor PMSF was added to a final concentration of 1 mM.

Primary purification of the target protein from the supernatant was carried out by chromatography on Ni-activated Sepharose (Cytiva, Marlborough, MA, USA). Then, the resulting protein was further purified on the SPS-Bio-SP sorbent (Technosorbent, Moscow, Russia). The eluted protein fraction was desalted to 150 mM NaCl and concentrated on an Amicon Ultra-0.5 column with a pore diameter of 10 kDa (Millipore, Burlington, MA, USA).

### 2.9. ELISA

#### 2.9.1. Indirect ELISA for Detection of Antibodies against the d34 Antigen in Serum

Indirect ELISA was performed according to the standard method [20] with minor modifications. In addition, the d34 antigen was adsorbed at a concentration of 2 μg/mL per well in a 96-well polystyrene plate (Corning #2592) in carbonate-bicarbonate buffer, pH 9.6, at 4 ℃ for 14 h. Thereafter, it was washed three times with phosphate-buffered saline with the addition of 0.02% Tween-20 at pH 7.2 (PBS-T). Plate blocking was performed with a 1% sodium caseinate solution for 2 h at RT. After the removal of the blocking solution, the plate was dried in a laminar box for 3 h. During ELISA, human or mouse sera, which had been previously diluted 100 times in PBS-T, were added to the wells of the plate. The incubation time was 60 min at 37 °C. Then, a standard procedure for detecting the amounts of antibodies bound to the d34 antigen was performed using an HRP conjugated anti-human IgG antibody (Bioservice, #M-2101-HRP) or anti-mouse IgG antibody (MP Biomedicals #55554). The optical density was measured spectrophotometrically at a wavelength of 450 nm on a Multiskan FC instrument (Thermo Scientific). The criterion for a positive result was an OD450 > 0.35.

#### 2.9.2. Indirect Sandwich ELISA for Detection of Antibodies against Different Serotypes of Dengue Virus in Mouse Serum

Indirect sandwich ELISA was performed according to the standard method [21] with minor modifications. Polyclonal rabbit antibodies against all 4 types of dengue virus (Bioservice) were adsorbed according to the standard protocol at a concentration of 10 µg/mL per well in a polystyrene plate (Corning #2592). Thereafter, antigens of each type of dengue virus were added to the wells of the plate at a concentration of 10 μg/mL in a volume of 100 μL. Antigens of various dengue serotypes were obtained from brain extracts of 4-day-old suckling mice infected intracranially with dengue viruses of various serotypes. The brain extracts were inactivated with β-propiolactone and clarified in a centrifuge. The brain extracts of mice not infected with the dengue virus were added to the control wells in the same volume. The plate was incubated at 37 °C for 60 min, then washed three times. When conducting ELISA, dilutions of mouse sera were preliminarily prepared with a dilution step of 2 from 1:100 to 1:3200 with PBS and 0.01M Tween20. Serum dilutions were added to the plate wells in triplicate for each serotype and control and incubated for 60 min at 37 °C. Then, the wells were washed three times with PBS-T, and a peroxidase conjugate of goat antibodies against mouse IgG (MP Biomedicals #55554) was added, followed by incubation at 37 °C for 60 min.

The optical density was measured spectrophotometrically at a wavelength of 450 nm on a LisaScan instrument (Erba Mannheim, Mannheim, Germany). The criterion for a positive result in wells with specific dengue antigen was an OD450 > 0.35.

### 2.10. Neutralization Test

The neutralization of the dengue virus with immune serum was carried out via the previously described method with some modifications [22]. The sera were inactivated at 56 °C for 30 min. Serum dilutions were prepared from 1:8 to 1:512 in increments of 2 in Leibovitz’s medium. One hundred microliters of each dilution were mixed with 100 µL of DENV2 containing 50 TCID50, followed by incubation for 1 h at 37 °C. Thereafter, the mixture was transferred to a monolayer of C6/36 cells in a well of a 24-well plate, followed by incubation at 32 °C with 5% CO_2_ for 5 days. The cytopathic effect (CPE) was observed using a microscope. On the 5th day, the culture fluid was sampled, and the presence of the viral nucleic acid in the medium was determined using the AmpliSense Dengue-Virus-FL kit (H-2391-1, Central Research Institute of Epidemiology of Rospotrebnadzor).

### 2.11. Data Analysis

Statistical data processing was performed using Statistica 8.0 software. Differences between groups were considered statistically significant if the p parameter did not exceed 0.05 according to the Mann-Whitney U test.

## 3. Results

### 3.1. Construction of the d34 Antigen and a Vaccine Variant Based on It

**Figure 1:**
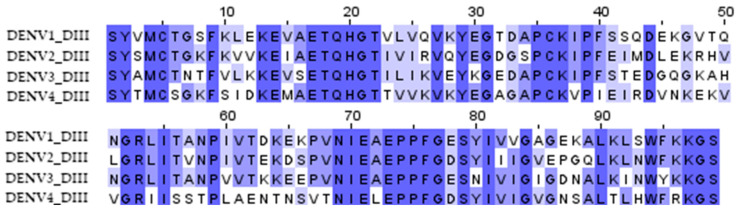
Multiple sequence alignment of the DIII domain amino acid residues of the dengue virus serotypes 1 (aa 578–676, record ID NP_059433), 2 (aa 578–676, record ID NP_056776), 3 (aa 576–674, record ID YP_001621843), and 4 (aa 577–675, record ID NP_073286) used to obtain the d34 antigen sequence. The color shows the similarity in the sequence.

**Figure 2:**
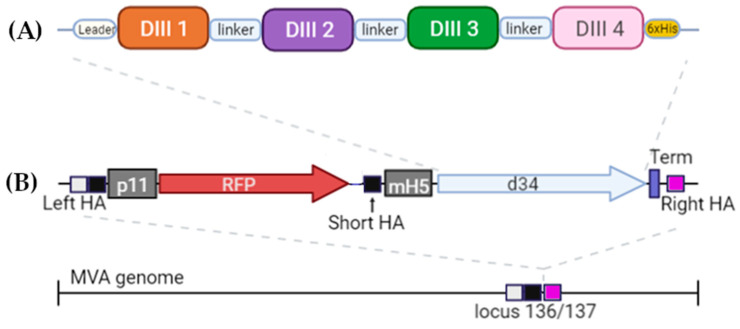
Schematic of the structure (**A**) of the antigen d34 and (**B**) expression cassette located in the MVA genome. Leader—the mouse Igk leader; linker—2x(GGGGS). The light box with the black box is the left homology arm (Left HA), the black box is the short left homology arm (Short HA), and the pink box is the right homology arm (Right HA). Gray boxes represent p11 and mH5 promoters from the vaccinia virus, driving the expression of RFP and d34 antigens, respectively. Vaccinia virus termination signal TTTTTNT (labeled as Term on the scheme) was used for stopping the transcription of RFP and d34 ORFs. More details can be found in Section 2.2.

The DIII domains of the E protein of the dengue virus were used to create the antigen since it had previously been shown that antibodies against the DIII domains of the E protein are serotype-specific, confer protection against homologous infection, and do not cause an enhancement of infection during heterologous infection [11,23]. A significant variation in the sequences of DIII domains of different serotypes probably explains the serotype specificity (Figure 1). To induce immunity against all four dengue virus serotypes, we combined the DIII domains of the 4 serotypes into a single amino acid sequence using flexible 2x(GGGS) linkers; the resulting protein was named d34 (Figure 2A).

In order to deliver the antigen, we used a vector based on MVA. An expression cassette containing the d34-coding gene under the control of the mH5 promoter together with the RFP reporter gene under the control of the p11 promoter was inserted into the 136/137 MVA locus (Figure 2B). More details can be found in Materials and Methods (Section 2.2).

### 3.2. Analysis of d34 Antigen Expression

**Figure 3:**
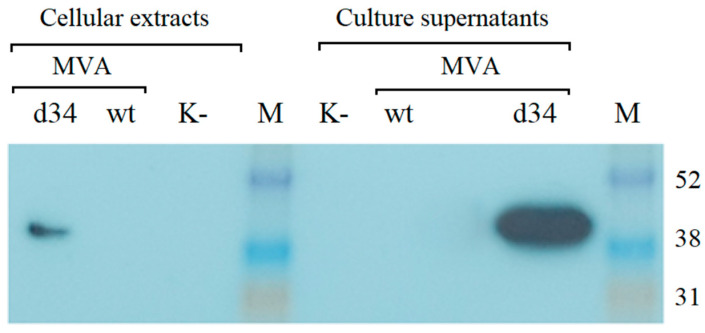
Western blot analysis of d34 antigen expression in BHK-21 cells after infection with the MVA-wt virus or recombinant rMVA-d34 virus (MOI = 2) using anti-6x-His Tag antibodies for detection. M is a marker of the molecular weight of proteins. K- indicates uninfected BHK-21 cells. The molecular weight of the d34 protein is 48.3 kDa. M-ECL™ Rainbow™ marker.

The recombinant rMVA-d34 virus containing the d34 gene at the 136/137 MVA locus was generated, purified, and amplified as described in the Section 2. The expression of the d34 gene in BHK-21 cells infected with rMVA-d34 was assessed by Western blot analysis using anti-6x-His Tag antibodies. One band corresponding to the expected protein size (48.3 kDa) was detected. Moreover, the protein was detected in both the cells and the growth medium, where its concentration was higher (Figure 3). Thus, the protein was produced in the cells and successfully secreted into the culture medium.

### 3.3. Study of the Interaction of the d34 Antigen with Human Blood Sera Containing IgG Antibodies against DENV, TBEV, and YFV Flaviviruses

The reactivity of the d34 protein with sera obtained from convalescent dengue individuals was studied via in-house indirect ELISA. This ELISA kit was designed to detect antibodies in the blood serum that can interact with the d34 protein, which is adsorbed on the solid phase. The results of the ELISA are presented in Table 1.

Additionally, to check for the absence of cross-reactivity of the d34 protein with antibodies against related viruses from the *Flaviviridae* family, sera obtained from people vaccinated against TBEV (10 sera) and YFV (10 sera) were also analyzed using in-house indirect ELISA (Table 1).

As follows from the data presented in Table 1, only sera containing IgG against the dengue virus interact with the d34 protein. Sera containing antibodies against tick-borne encephalitis or yellow fever virus do not react with the d34 antigen.

In addition, the specificity of recognition of the d34 protein by IgG antibodies from different sera was assessed using Western blot analysis. d34 gene expression was performed by infecting HEK293FT cells with the rMVA-d34 virus, and wt MVA-infected or uninfected cells were used as negative controls. Samples of the culture medium were collected and analyzed by Western blotting by interacting with different human sera or with anti-6x-His Tag antibodies.

**Figure 4:**
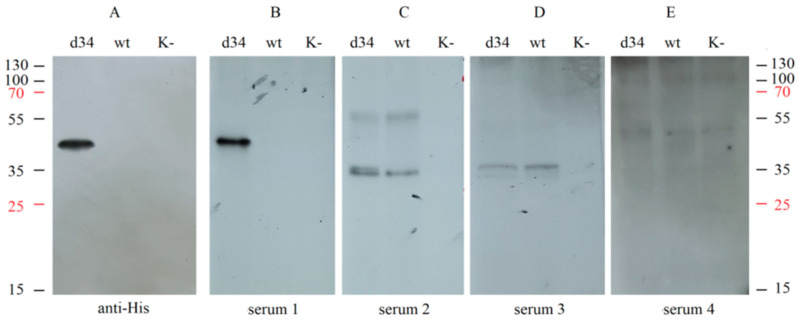
Analysis of the interaction of the d34 antigen with blood sera using Western blotting. HEK293FT cells were infected with MVA (wt), recombinant MVA-d34 (d34), or mock (K−). The d34 antigen was probed with (**A**) anti-6x-His Tag antibodies (anti-His); (**B**) dengue fever-positive human sera (serum 1); (**C**) human sera from a TBEV-vaccinated individual (serum 2); (**D**) human sera from a TBEV- and YFV-vaccinated individual (serum 3); and (**E**) human sera from a YFV-vaccinated individual (serum 4). The numbers on the sides correspond to the protein molecular weight marker (M-PageRuler™ Plus Prestained Protein marker). K cells, HEK. The weight of the d34 protein is 48.3 kDa.

Hybridization with the sera from convalescent dengue patients resulted in a single band at the same level as the anti-6x-His Tag antibody (Figure 4A,B); i.e., the d34 antigen was specifically recognized by sera containing antibodies against dengue virus. At the same time, hybridization with human serum containing antibodies against TBEV (serum 2), TBEV, and YFV (serum 3), or only YFV (serum 4) did not result in a band corresponding to the d34 protein (Figure 4C–E). This indicates the absence of cross-interaction of the d34 antigen with antibodies against YFV and TBEV.

Notably, the two sera tested, serum 2 and serum 3, showed a similar pattern when hybridized with wt MVA-infected and rMVA-d34-infected cells but not with uninfected cells, suggesting that these sera contain antibodies against MVA proteins. Such antibodies most likely appeared because the sera were obtained from people who were born before 1980 and therefore received the smallpox vaccine, which has many antigens in common with MVA.

Taken together, these results show that the d34 protein binds to antibodies against the dengue virus but does not bind to antibodies against closely related viruses from the flavivirus family (TBEV, YFV). This indicates that the recombinant d34 protein, which consists of four DIII domains of the E protein belonging to different serotypes of the dengue virus, shows antigenic specificity.

### 3.4. Evaluation of the Immune Response after Vaccination of MVA-d34 Mice

**Figure 5:**
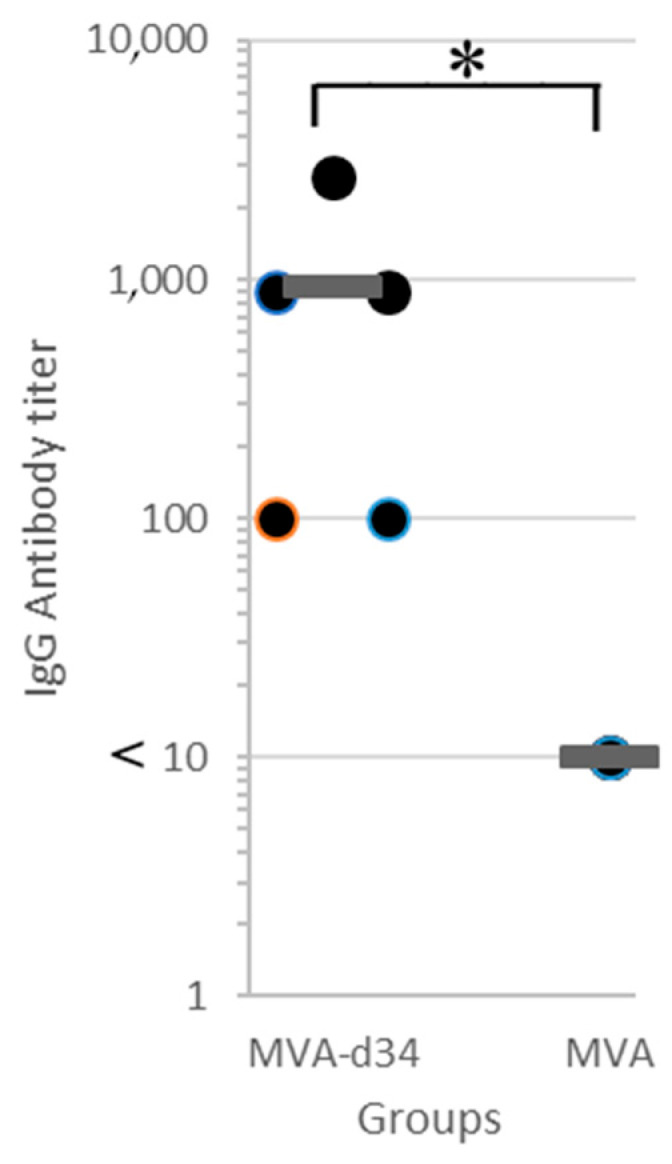
Antibody titer in blood sera of mice immunized with MVA-d34 (N = 5) or wt MVA (N = 5) measured via in-house indirect d34 ELISA. The mean value is shown by a thick line. The asterisk denotes a statistically significant difference between MVA-d34 group and wt MVA control group (*p*-value < 0.05 according to the Mann-Whitney U test).

The resulting recombinant variant of MVA-d34 was used to vaccinate mice. One group of mice (N = 5) was immunized twice with MVA-d34, and the second group was injected with wt MVA (control group, N = 5). Three weeks after the second immunization, blood sera were obtained from all animals, and their antibody titers were determined via in-house indirect ELISA (Figure 5).

For titer quantification, the sera were three-fold serially diluted with PBS-T from 100 to 2700. The criterion for a positive result was an OD450 > 0.35. An antibody titer value of 1:2700 was obtained for one serum sample, 1:900 for two, and 1:100 for two. At the same time, in all sera obtained from mice in the control group at a dilution of 1:10, the OD450 value did not exceed 0.25. Thus, all mice (N = 5) immunized with MVA-d34 showed an immune response to the d34 antigen.

### 3.5. Evaluation of the Immune Responses against Different Dengue Virus Serotypes after Vaccination with MVA-d34

The d34 antigen was designed to elicit a humoral immune response against all four dengue virus serotypes. Sera from three immunized mice were tested for antibodies against each dengue virus serotype using an in-house indirect sandwich ELISA test.

As shown in Table 2, all tested sera interacted with each of the 4 dengue viruses serotypes at dilution limits ranging from 1:400 to 1:800. Sera from mice vaccinated with wt MVA did not interact with any of the serotypes (Table 2).

Thus, after immunization with the recombinant MVA-d34 viral vector, mice produced specific IgGs that interacted with the antigens of all four dengue virus serotypes.

### 3.6. Assessment of the Neutralizing Activity of Blood Sera from Mice Immunized with MVA-d34

The virus-neutralizing activity of the sera of mice immunized with MVA-d34 against dengue virus serotype 2 was studied in a C6/36 cell culture. Sera from three mice with higher antibody titers measured by ELISA were pooled for the study. The addition of sera obtained from non-vaccinated or vaccinated mice without virus infection (negative control for serum) at a dilution of 1:8 did not have a cytopathic effect on C6/36 cells throughout the observation period. Pure virus and virus mixed with serum from a non-vaccinated mouse were used as positive controls for viral infection. The observation results are presented in Table 3.

When cells were infected with the virus without the addition of serum, disruption of the monolayer was observed at a rate exceeding 75%. Serum obtained from a non-vaccinated animal showed no virus-neutralizing activity; the cytopathic effect was greater than 75%, and the PCR results were positive. In contrast, sera from mice immunized with MVA-d34 showed a virus-neutralizing effect at dilutions up to 1:64; at these dilutions, no cytopathic effect of the virus on the cells was noted, and the PCR results were negative.

## 4. Discussion

The humoral immune response in individuals who have recovered from infection with one of the dengue virus serotypes is cross-reactive against other dengue virus serotypes. However, the response related to heterologous strains is much weaker and does not protect recovered people from infection with strains of other serotypes. Moreover, such incomplete cross-reactivity often leads to ADE [24]. When vaccination is performed with the full-length surface protein E, a similar problem arises. Thus, in the clinical trials of the Dengvaxia vaccine, it was shown that vaccination of previously ill patients may increase the risk of developing ADE [3,4,5]. In recent years, the DIII domain of the E protein has been used as an antigen, which does not produce cross-reactive antibodies that cause ADE [25].

Previously, several different platforms have been used to deliver the DIII antigen. The administration of DNA vaccines via intramuscular injection was found to induce a weak antibody response [26]. Later, a pronounced immune response was achieved due to attachment to the DIII domain of the CH3 domain from human IgG, which enhanced the production of the DIII antigen [8,9]. In these works, a mixture of antigens from all four serotypes was delivered in the form of coding plasmids on gold particles (GeneGun technology, Bio-Rad). In another study, a tetravalent vaccine containing a mixture of four recombinant fusion proteins, consisting of the DIII domains of each dengue virus serotype and the p64k immunogenic domain from meningococcus [10], also elicited a good antibody response. However, despite the presence of an immunogenic domain from meningococcus, the authors additionally used an adjuvant (aluminum hydroxide), which may indicate weak immunogenicity of the antigen itself. In the other two studies, an antigen consisting of the DIII domains of all four serotypes connected in a single chain was used [11,12]. In one case, a live attenuated measles vector vaccine was used for delivery [12]. The authors showed the production of antibodies against all serotypes in response to vaccination, but the neutralizing activity of the antibodies was very weak. In another case, when a similar antigen was delivered using virus-like particles based on the hepatitis B S protein with the addition of an adjuvant (aluminum hydroxide), a good immune response protecting animals from viral infection was achieved [11].

In our work, we used the viral vector MVA, whose safety has repeatedly been shown in clinical trials [13] and during mass vaccination against smallpox in Germany [27,28]. The safety and high immunogenicity of the MVA vector have also been demonstrated in the vaccination of immunocompromised people (for more details, see review [13]). In addition, MVA-based vaccines induce a good T-cell response, which may be important for protection against flaviviruses. Thus, data on the important role of the T-cell response in protection against yellow fever [29] and chikungunya [30] viruses were obtained.

When constructing the antigen, an approach similar to that used previously [11,12] was employed. To obtain the antigen (d34), the DIII domains of all four dengue virus serotypes were combined into a single chain; however, in contrast to the previously described approaches, we used flexible 2x(GGGS) linkers to connect the domains. This made it possible to create an antigen that stimulates the immune responses against four serotypes of the dengue virus at once and, on the other hand, to achieve high gene expression since it is known that single domains are poorly expressed in cells [9].

Furthermore, to study the specificity of the obtained d34 antigen, its interaction with sera from individuals who had recovered from dengue fever or individuals vaccinated against TBEV and YFV was analyzed using ELISA tests and Western blotting. Sera from vaccinees who were confirmed to have antibodies against TBEV and YFV were of interest for two reasons. First, all three viruses often circulate in the same regions. Second, they are closely related viruses from the flavivirus genus that often show cross-reactivity in ELISAs. However, the d34 antigen has been shown to react with convalescent dengue sera and not with sera from individuals vaccinated against other flaviviruses.

The immunogenic characteristics of the MVA-d34 vaccine were evaluated by immunizing mice. All immunized animals developed high titers of antibodies against the dengue virus antigen. A detailed study of the breadth of the immune response showed that mice form specific IgGs that interact with the antigens of all four dengue virus serotypes. Moreover, the neutralizing activity of sera at a dilution of 1:64 against dengue virus serotype 2 was shown in cell culture.

Thus, in our work, we constructed and characterized the d34 dengue virus antigen and showed that when it is delivered using the MVA viral vector, this antigen causes a humoral response in mice against all dengue virus serotypes without cross-reactivity with antigens of closely related viruses from the flavivirus family (TBEV, YFV). In addition, the developed immunity exerts virus-neutralizing activity toward dengue virus serotype 2. The developed candidate vaccine MVA-d34 shows promise for further studies of its use as a vaccine against dengue fever in humans.

## Figures and Tables

**Table 1:** Results of studying the reactivity of the d34 antigen with human blood sera containing IgG antibodies against DENV, TBEV, and YFV. For each group of sera, the number of positive and negative samples identified using the corresponding ELISA test is indicated. The ranges of OD450 values for all sera from this group are shown in brackets.

	ELISA Tests	d34 ^1^		DENV ^2^		TBEV ^3^		YFV ^4^	
Human Sera		+	−	+	−	+	−	+	−
Recovered from dengue fever		7 (1.244–4.000 *)	0	7 (0.856–2.482)	0	0	7 (0.006–0.134)	0	7 (0.082–0.150)
Vaccinated against TBEV		0	10 (0.096–0.135)	0	10 (0.074–0.102)	10 (0.676–4.000)	0	0	10 (0.093–0.158)
Vaccinated against YFV		0	10 (0.058–0.126)	0	10 (0.066–0.137)	0	10 (0.024–0.110)	10 (0.867–1.256)	0

1—In-house indirect ELISA based on the d34 antigen (see methods 2.9.1); 2—Anti-Dengue Virus ELISA (IgG) (Euroimmun AG, Germany); 3—VectoTBEV-IgG (AO Vector-Best, Russia) D-1156; 4—Qualitative Human Yellow Fever Virus Antibody IgG (YFV-IgG) ELISA Kit. *—The LisaScan (Erba Mannheim) has an upper limit of the optical density measurement range at 4 OE.

**Table 2:** The results of the analysis of blood sera of mice immunized with MVA-d34 and mice immunized with wt MVA for the presence of antibodies against different dengue virus serotypes using the in-house indirect sandwich ELISA test.

Mice		Serum Titer with OD > 0.35			
		Dengue Virus Serotype			
		1	2	3	4
Immunized by MVA-d34	1	1:800	1:800	1:800	1:400
	2	1:800	1:800	1:800	1:800
	3	1:800	1:800	1:400	1:400
Immunized by wt MVA		n.d. *	n.d.	n.d.	n.d.

* n.d.—not defined, OD less than 0.2.

**Table 3:** The results of the study of the virus-neutralizing activity of blood sera from mice immunized with MVA-d34 and non-vaccinated mice.

Serum Used for Incubation with the Virus	Serum Dilution	Detection of a Cytopathic Effect of the DENV2 Virus	Virus Detection by qPCR
From mice immunized with MVA-d34	1:16	No	Negative
	1:32	No	Negative
	1:64	No	Negative
	1:128	Yes	Positive
	1:256	Yes	Positive
	1:512	Yes	Positive
From a non-vaccinated mouse	1:8	Yes	Positive
No sera	-	Yes	Positive

## Data Availability

Data supporting reported results can be requested from the authors.

## Supplementary Materials

The following supporting information can be downloaded at: https://www.mdpi.com/article/10.3390/vaccines11040831/s1.
