# Supplementary material for: Development of MVA-d34 Tetravalent Dengue Vaccine: Design and Immunogenicity
Source: Vaccines (Basel). 2023 Apr 12;11(4):831. doi: 10.3390/vaccines11040831 (PMC10142911; doi:10.3390/vaccines11040831)
Supplement: Supplementary file 1 [file vaccines-11-00831-s001.zip › vaccines-2319925-supplementary.pdf]

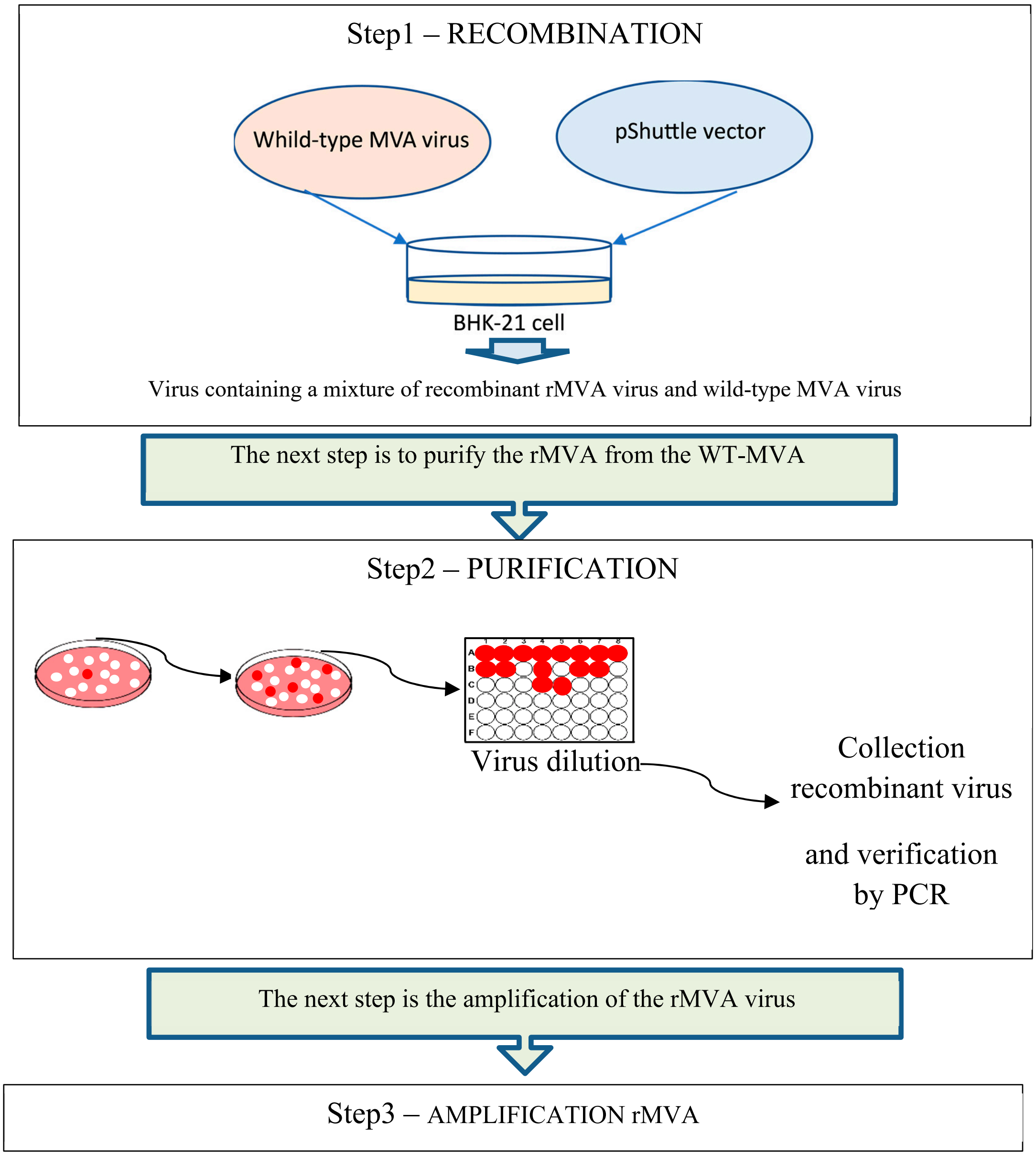

Figure S1. Scheme of the method used to generate rMVA. In Step 1, recombinant vaccinia virus was constructed through homologous recombination in BHK-21 cells that were first infected with wild-type MVA virus and then transfected with pShuttle vector. This resulted in a mixture of wild-type and recombinant MVA virus. In Step 2, individual colonies were isolated on a 6-well plate and the recombinant MVA virus was purified from the wild-type virus, followed by post-purification in a 48-well plate. Finally, in Step 3, the recombinant MVA virus was amplified in a T175 flask.

Table S1. Sequences of amino acid residues of the DIII domain from the four dengue virus serotypes used in this study.

>NP_059433 DENV1_DIII (aa 578-676) SYVMCTGSFKLEKEVAETQHGTVLVQVKYEGTDAPCKIPFSSQDEKGVTQNGRLITANPIVTDKEKPVNIEAEPPFGESYIVVGAGEKALKLSWFKKGS
>NP_056776 DENV2_DIII (aa 578-676) SYSMCTGKFKVVKEIAETQHGTIVIRVQYEGDGSPCKIPFEIMDLEKRHVLGRLITVNPIVTEKDSPVNIEAEPPFGDSYIIIGVEPGQLKLNWFKKGS
>YP_001621843 DENV3_DIII (aa 576-674)

SYAMCTNTFVLKKEVSETQHGTILIKVEYKGEDAPCKIPFSTEDGQGKAHNGRLITANPVVTKKEEPVNIEAEPPFGES NIVIGIGDNALKINWYKKGS
>NP_073286 DENV4_DIII (aa 577-675) SYTMCSGKFSIDKEMAETQHGTTVVKVKYEGAGAPCKVPIEIRDVNKEKVVGRIISSTPLAENTNSVTNIELEPPFGDS YIVIGVGNSALTLHWFRKGS

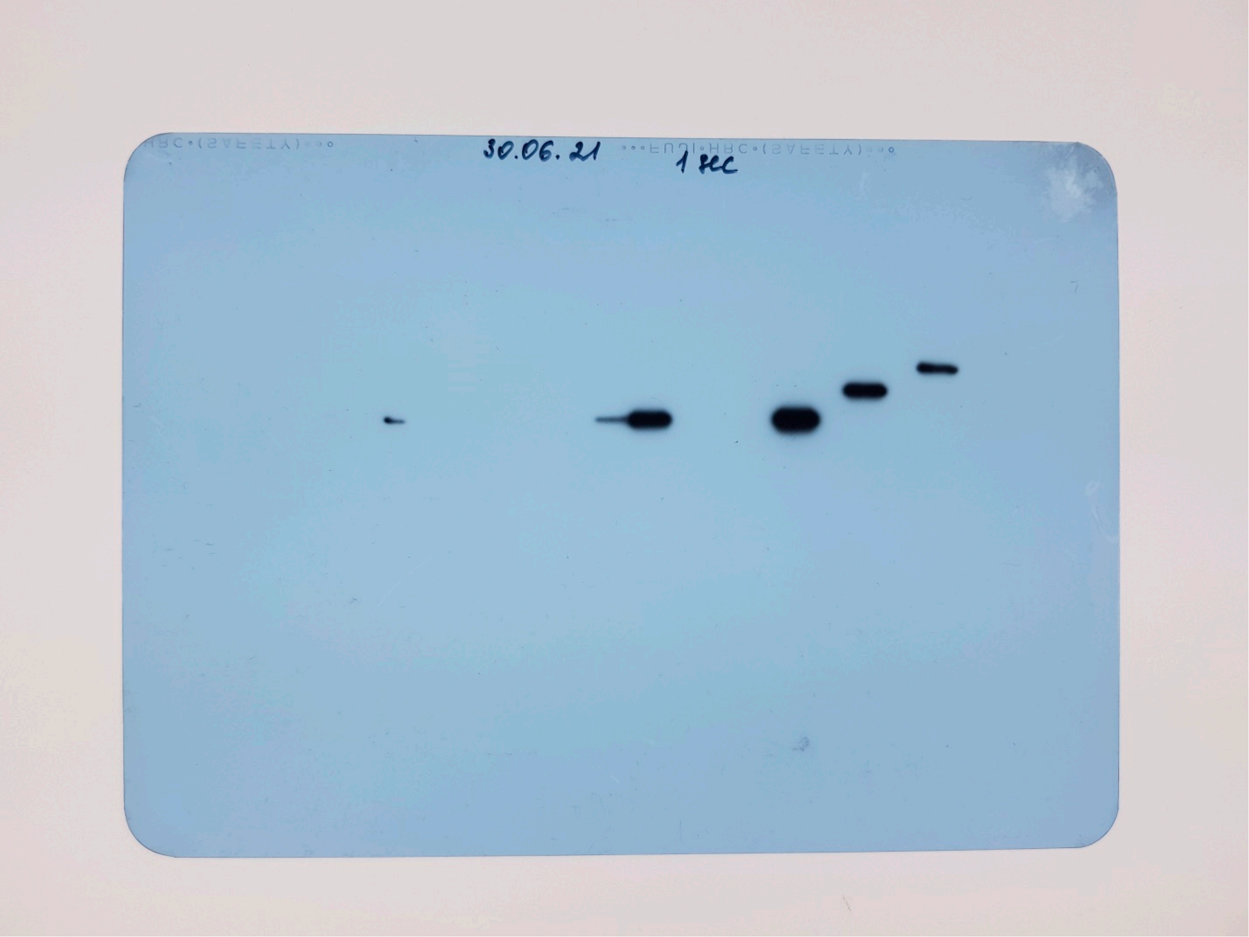

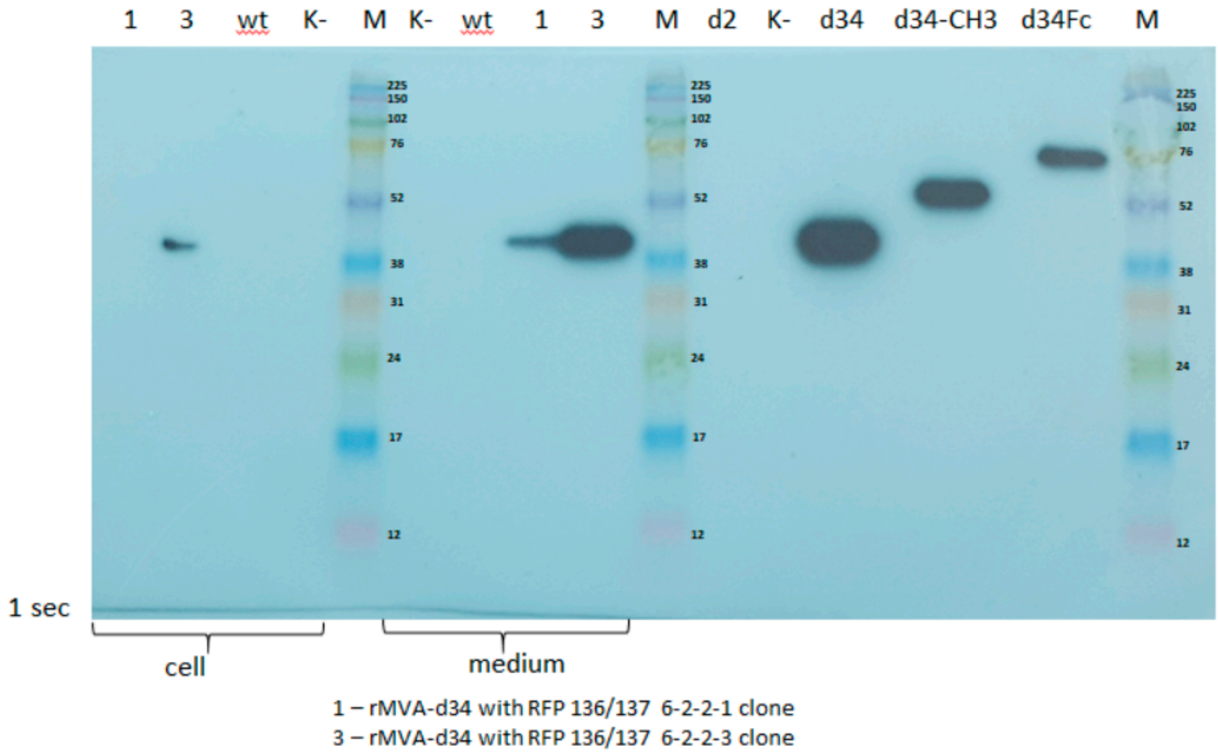

Figure S2.Film and membrane to Figure 2.

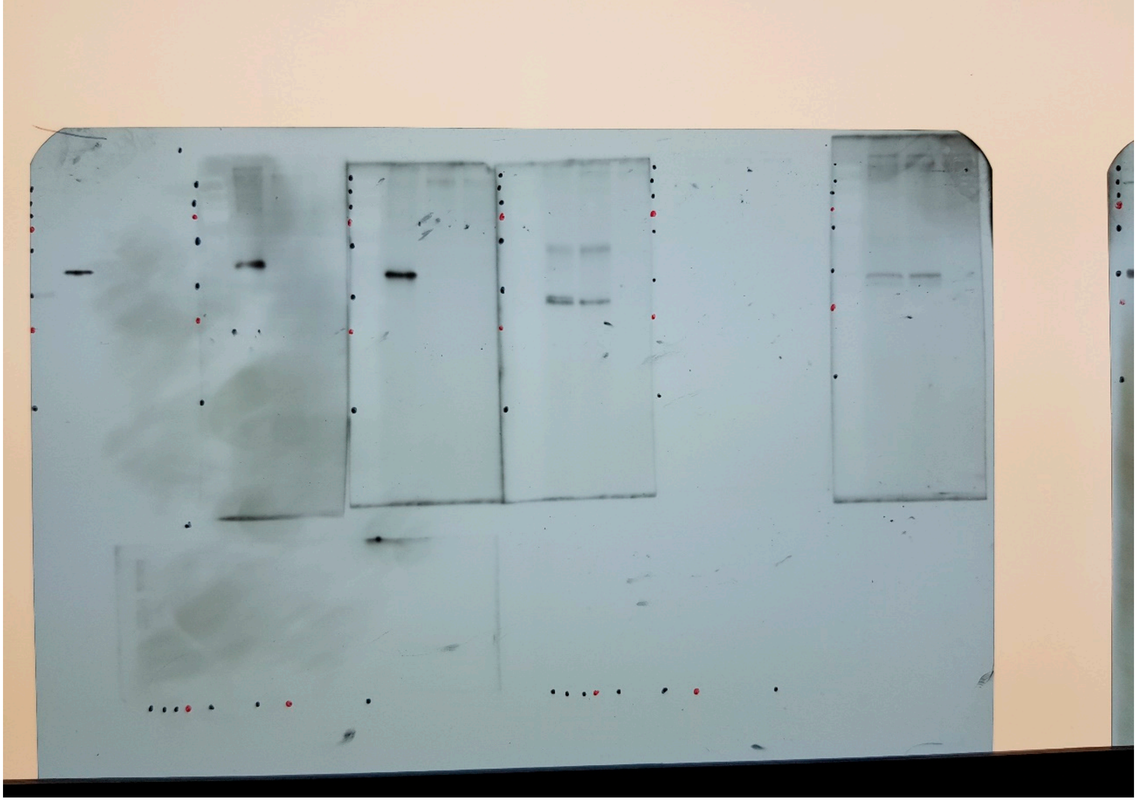

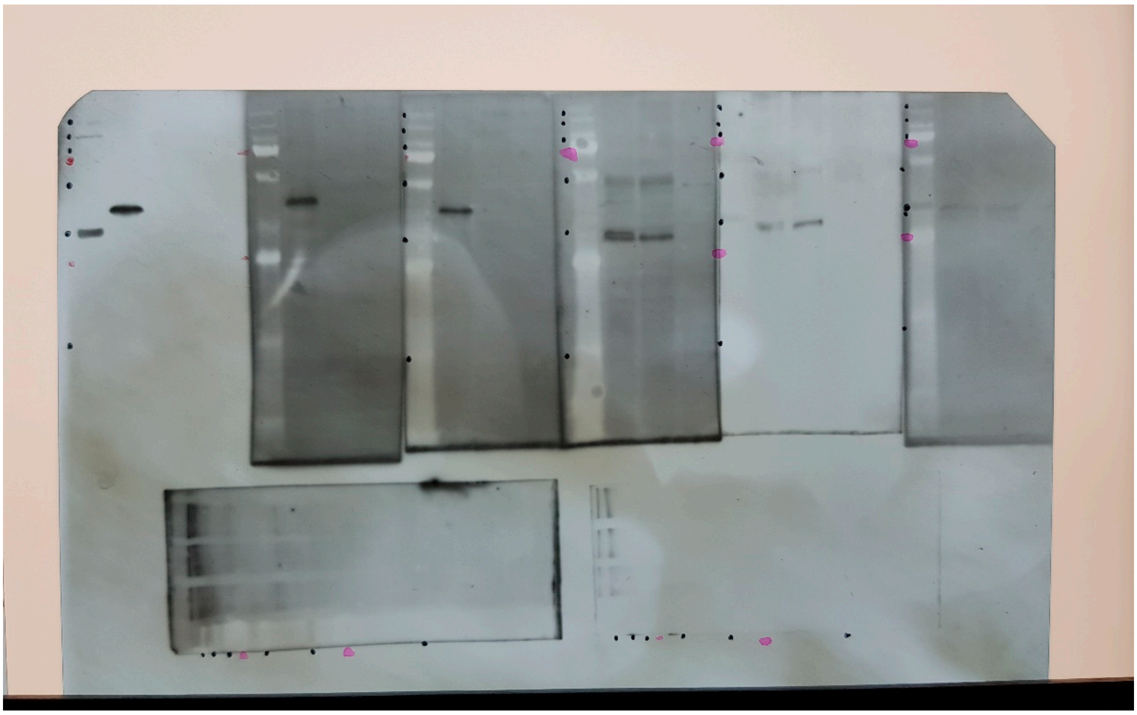

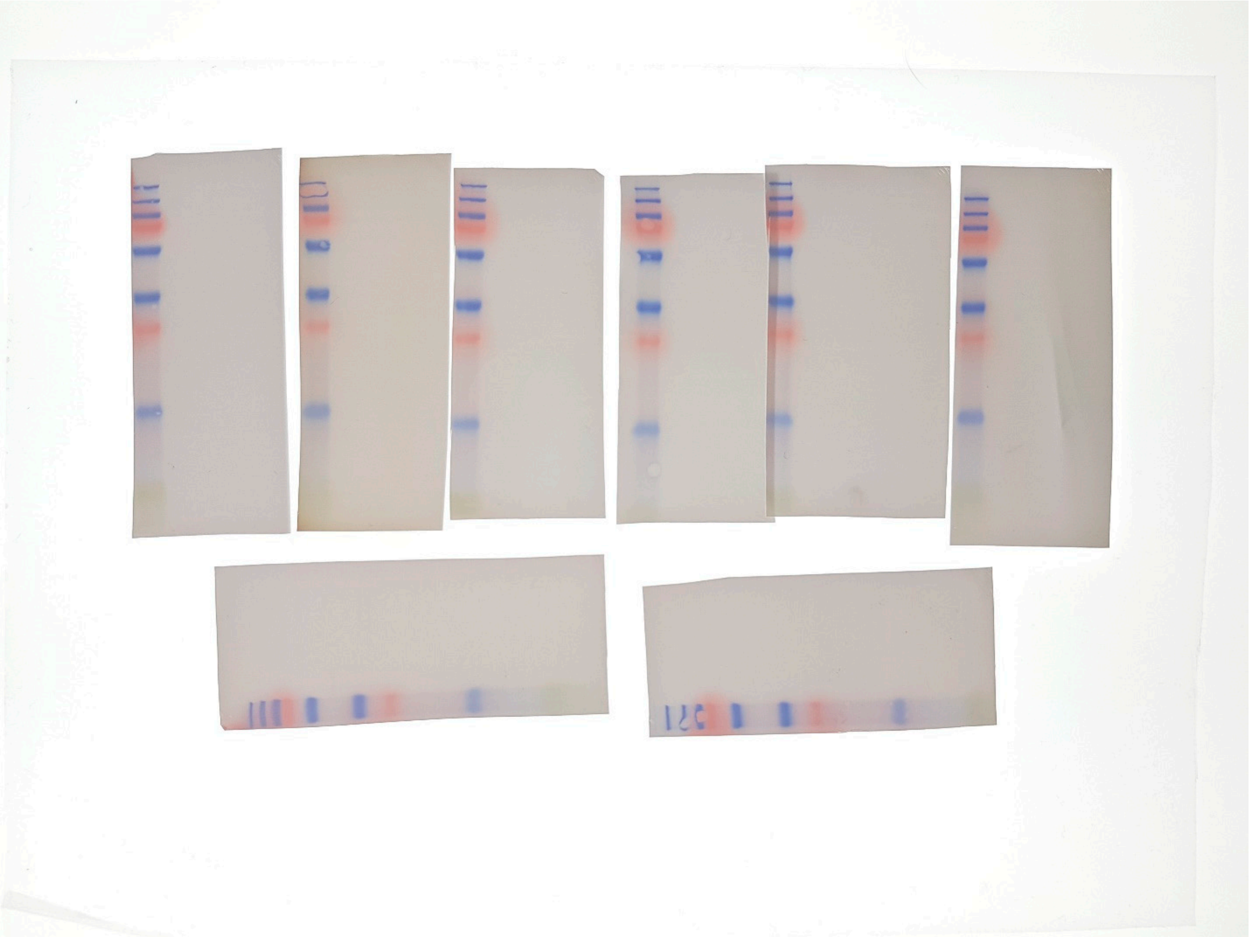

Figure S3.Film and membrane to Figure 3.
